# Automated Psychotherapy in a Spaceflight Environment: Advantages, Drawbacks, and Unknowns

**DOI:** 10.2196/58803

**Published:** 2024-10-09

**Authors:** Logan Smith

**Affiliations:** 1 Oklahoma State University Stillwater, OK United States

**Keywords:** mental health, deep space, astronauts, aerospace medicine, spaceflight, flight, psychotherapy, privacy, communication

## Abstract

Various behavioral and mental health issues have been reported by space crews for decades, with the overall number of mental health complications expected to be higher than is publicly known. The broad range of mental health complications encountered in space is expected to grow as people venture deeper into space. Issues with privacy, dual relationships, and delayed communications make rendering effective psychological therapy difficult in a spaceflight environment and nearly impossible in deep space. Automated psychotherapy offers a way to provide psychotherapy to astronauts both in deep space and low Earth orbit. Although automated psychotherapy is growing in popularity on Earth, little is known about its efficacy in space. This viewpoint serves to highlight the knowns and unknowns regarding this treatment modality for future deep space missions, and places an emphasis on the need for further research into the applicability and practicality of automated psychotherapy for the spaceflight environment, especially as it relates to long-duration, deep space missions.

## Introduction

The assumed importance of mental health in a spaceflight environment has changed over time, with earlier mission planners thinking that mental health complications would not be an issue for sufficiently qualified personnel [1]. This “right stuff” mentality was thought to indicate that astronaut candidates who were resilient to stress, capable of operating under extreme pressures, and able to draw on a reserve of inner strength to push through dangerous and anxiety-provoking circumstances would be the ideal astronaut in space [2]. However, these highly specific selection criteria do not appear to be capable of picking individuals who are impervious to mental health complications during spaceflight, with a number of notable examples being reported over the past 60 years.

In 1982, a Russian cosmonaut reported struggles with isolation and depressed mood while in space [3]. A 1985 Russian Soyuz mission ended 60% earlier than expected due to what is believed to be issues involving “mood and performance” for crew members [3]. In addition, over the course of 89 American space shuttle missions, there were 34 separate behavioral issues reported among the 208 different crew members [4]. The differing labels used for these instances—hostile and uncooperative, depressed mood, mood and performance, and behavioral issues—reflect the imprecise way in which mental health complications have been discussed in relation to astronaut activities. It is important to note that these instances do not necessarily reflect something that would have threatened the safety of crew or the mission and were often transient.

Of the psychological and behavioral problems that have been reported and made public, recorded issues include anxiety, depression, irritability, sleep-wake disorders, asthenia, interpersonal tension, impaired judgement, inappropriate behavior, stress, exhaustion, euphoria, neurosis, accentuation of negative personality traits, and various cognitive impairments [4-7]. While these reported psychological and behavioral problems are broad, it should be noted that these have all been reported in missions to low Earth orbit; it is not yet determined what psychological issues could present during long-duration, deep space missions. A general consensus is that all of these previously reported problems could occur in deep space, in addition to serious psychiatric symptoms, psychotic disorders, delirium, and homesickness, among others [4,8-10]. Although these examples are noteworthy, they do not give a complete picture of the mental health incidences that have occurred during spaceflight. It is believed that the actual incidence rate is higher than previously reported and that a number of astronauts may be unwilling to divulge these details with ground personnel [11]. The purpose of this viewpoint is to summarize the current state of automated psychotherapy for the spaceflight environment, especially as it relates to the advantages that it offers, the drawbacks associated with this treatment modality, and the current unknowns.

## Current Treatments for Mental Health Problems in Space

Mental health interventions in a spaceflight environment begin at the crew selection phase, where astronauts are screened for commonly known psychological risk factors [2], commonly referred to as “select-out” logic. In addition, in accordance with “select-in” logic, previous research has indicated that the ideal psychological profile for a crew member for a space mission, especially a long-duration, deep space mission, is an individual with high levels of adaptability, resistance to stress, psychological stability, and strong social skills [12]. It is believed that these highly specific psychological selection criteria may help reduce the rate of psychological issues experienced during the mission. In addition, the Human Behavior and Performance Support Program, a component of NASA comprised of highly trained psychologists, physicians, and other mental health professionals, is tasked with providing support to an astronaut at all phases of the mission—before, during, and afterward. The Human Behavior and Performance Support Program offers entertainment and activities to counteract the detrimental effects of boredom and isolation, regular private video conferences with psychologists on the ground, interactions with family and friends, and other supportive measures to prevent mental health complications from escalating to a degree that may present problems for mission success and could require further treatment.

When a mental health complication is reported during a spaceflight mission, ground personnel, medical personnel, and astronauts have a variety of methods available to them to treat the condition. The first step often focuses on reducing the levels of stress that the astronaut is feeling, as the very environment of a spaceflight mission may produce physical and psychic strain on the astronaut through a combination of microgravity, noise, isolation, reduced privacy, radiation, work-leisure balance, and other factors [1,7,10,11,13,14]. While stress reduction is commonly used as a preventative measure, it is also helpful after symptoms of psychopathology have been reported [8]. One research team has categorized the general stress reduction strategies for a spaceflight environment to show their focus on 4 main areas: ergonomic (factors associated with the design of the spacecraft), physiological (factors associated with nutrition, sleep, and hormonal balance), psychological (factors associated with known psychological stressors), and psychosocial (factors associated with relationships between the astronaut and other crew members as well as ground personnel) [14]. Recommended treatments for dealing with stress while in space often involve increased leisure time, increased time spent communicating with loved ones on Earth, and structured interventions. One such structured intervention is the Spaceflight-Induced Stress Management Plan, which involves a series of group training modules that prepare astronauts for stressors and equips them with the skills needed to form support groups while in space [14]. Given the strong connection between reduced coping with stress and subsequent psychopathology [15], it is evident that the ability to de-escalate the stress that an astronaut is feeling may reduce their likelihood of developing mental health complications, symptoms, and disorders while in space. However, it is important to note that while stress reduction initiatives can help improve the overall mental health of an astronaut, they are not likely to be a sufficient intervention to treat psychopathological responses, such as depression, psychosis, or severe anxiety disorders.

In the event that a mental health complication has been reported that cannot be remedied by noninvasive stress interventions alone, it is recommended that some sort of psychological therapy begin [6]. Regular scheduled interactions between astronauts and a psychologist on the ground may provide a normalized way for crew members to discuss their emotions, relieve tension, and receive feedback from trained specialists [16]. However, the usual version of psychological therapy used on Earth, where a provider and a client have roughly 1 hour of uninterrupted, private, structured discussions using a standardized treatment, is exceedingly difficult in space, and there are little available data on the practice or outcomes of psychological therapy in a spaceflight environment. This will be discussed in further detail in the *Difficulties of Therapy in Space* section. In addition, the use of monitoring tools that can be used to capture intraindividual variability across various behavioral and mental health markers, including real-time physiological measurements and medical monitoring, should be explored as a way to passively and actively gather data on the current functioning of astronauts as a way of detecting potential mental health concerns as early as possible. Often, these tools are used in psychological research to produce reliable data and can involve nonintrusive wearable devices to detect heart rate and skin conductance (such as a ring or a wristband), as well as passive environmental monitors for eye tracking and movement.

A commonly used treatment for mental health complications in space is medication, which is a separate treatment modality from psychotherapy and may be used independent of psychotherapy or in conjuncture with psychotherapy. The current psychiatric formulary onboard the International Space Station includes antidepressants, antipsychotics, anxiolytics, anticholinergics, sleep agents, and wake agents [4]. It is important to note that these medications are helpful for treating the expected psychological disorders that may be encountered in low Earth orbit and do not include medications for more severe psychopathologies, such as more powerful antipsychotics. Rather, this formulary was created with the assumption that severe psychological emergencies may be treated by a rapid return to Earth. Future deep space missions may need a more comprehensive formulary for a setting where a return to Earth is not feasible [4].

Taken together, it is apparent that most interventions for mental health complications in a spaceflight environment have focused on crew selection criteria and methods for coping with stress. While psychiatric medications are available in space and have been used by at least 24 crew members spread out among 20 different missions [17], the efficacy of the gold standard of psychological care, structured psychological therapy, is largely unknown for the spaceflight environment. The following section will focus on this topic.

## Difficulties of Therapy in Space

It is recommended that physicians aboard the International Space Station be trained in psychological therapy in order to provide direct psychological care to crew members with a psychological disorder or symptom [18]. However, the American Psychological Association’s Ethical Principles of Psychologists and Code of Conducts outline that a psychologist providing therapy to an individual, while at the same time already possessing another relationship with that person, is engaged in multiple relationships—a potentially dangerous state for a therapist and client to be in that represents real hazards to both individuals [19]. While dual relationships offer a potential pathway for harm to come to either the therapist or the client, they cannot always be avoided [20]. When one considers the perils associated with strained relationships in a spaceflight environment, including lack of cooperation between parties, interpersonal conflicts, reduced privacy and escape, and other hazards, it is apparent that any situation that could produce these hazards should be avoided wherever possible. For these reasons, it may be advisable to avoid providing psychological therapy to a crew member from another crew member on the same mission, if possible.

An alternative to intracrew psychological therapy may be ground-based psychological teletherapy. The proliferation of teletherapy during the COVID-19 pandemic has provided a way for more people to receive psychological care than was previously possible and has acted as an accelerator for this sort of technology [21], acting as a proving ground for the efficacy of teletherapy. However, the application of teletherapy to a spaceflight environment has not been fully explored. Astronauts in space commonly complain about a lack of privacy while onboard a spacecraft or space station [22,23], which may make teletherapy exceedingly difficult while in space. The lack of privacy during therapy, often referred to as a lack of a “safe therapeutic space,” can seriously threaten the effectiveness of psychological therapy [24], as a therapy client may be unwilling or unable to divulge crucial details, express emotions fully, or fully participate in therapy in other crucial ways.

Although a lack of privacy in a spaceflight environment can conceivably be corrected through modified living quarters, one issue that cannot be corrected is delayed communications. Previous space simulation studies have revealed that delayed communication creates a host of problems for crews, including confusion and wasted crew time, decreased verbal encoding efficacy, increased stress and frustration, and general task and communication errors [25-28]. Surprisingly, difficulties were seen across the range of possible delayed voice communication times, with issues being found when messages were delayed by as brief as just fractions of a second [26] or as long as 5 minutes [25]. Currently, crews aboard the International Space Station experience communication delays between the crew and the mission support team on the ground measured in the millisecond range, although it is known that this will increase to upwards of 22 minutes for crewed missions to Mars [29], and even longer for more distant destinations. It is apparent that humans are sensitive to voice communications being delayed by any noticeable amount, with difficulties, errors, and stress quickly appearing under these conditions; during an emotionally charged psychotherapy session, these effects may be more pronounced.

Although psychological therapy is considered to be one of the most desirable psychological interventions and is empirically supported for both short-term and long-term outcomes, its use in a spaceflight environment may be difficult. Dual relationships between crew members create barriers to intercrew psychotherapy by introducing increased risk for other negative outcomes. In addition, a combination of a lack of privacy and delayed communications creates significant barriers for teletherapy between ground personnel and astronauts in low Earth orbit. As such, there are limited options available for psychotherapy in low Earth orbit.

## Particular Issues Posed by a Deep Space Environment

A deep space mission may have particular stressors that create an increased risk for mental health complications; in addition to the known stressors of spaceflight (eg, monotony, microgravity, awareness of danger, interpersonal tension, and radiation), a deep space mission may encounter additional psychological stressors, including increased isolation, the psychological effects of distance from Earth, the knowledge of a lack of rescue, prolonged homesickness, and other related phenomena [10,30]. In addition, the very nature of deep space creates a certainty that delayed communications will become a facet of life for deep space astronauts; as the speed of light dictates the maximum speed at which information can travel, messages from Earth to Mars can take up to 22 minutes in 1 direction [29], with longer delays being a certainty for deeper space missions to asteroids, the moons of the gas giants, and other destinations. As such, delayed communication is expected to be one of the most pressing issues with regard to future long-duration, deep space missions [25]. When combined with the known and expected stressors associated with deep space missions, it is clear that an option for psychological care is needed that does not rely on Earth-based interventions, while also avoiding the dual relationship hazards of intercrew psychotherapy.

## Need for Automated Psychotherapy for Spaceflight

An increasingly common psychological treatment modality, automated psychotherapy, sometimes called computer-mediated psychotherapy, cybertherapy, or computer-created virtual reality for therapy [31,32], is often seen as a method of delivering psychological therapy (psychotherapy) to various groups of people, especially populations that are underserved or otherwise unreachable through traditional therapeutic routes [33]. Automated psychotherapy generally takes the form of a series of computer-delivered modules that explain applicable psychological terms to the therapy client, provides a degree of psychoeducation necessary to progress through the module, delivers homework tasks and the necessary training required for those tasks, and assesses the symptoms and progress of the client using standardized empirically supported measures. Automated psychotherapy is not to be confused with self-help books, which, although found to be effective for temporary amelioration of depressive symptoms (among other disorders), have not been found to produce lasting effects past 6 months [33]. Rather, automated psychotherapy has been shown to be highly effective in treating various psychopathologies with lasting and reliable results [34].

Automated psychotherapy provides a way for astronauts to receive empirically supported psychological therapy while in space and distant from the Earth. Notably, as will be outlined in greater detail below, automated psychotherapy is able to mitigate the issues that confound traditional therapy or teletherapy in a spaceflight environment. There are research projects currently underway to investigate the efficacy of, and design a treatment for, automated psychotherapy in a spaceflight environment [35]; these findings will be explored in greater detail below. Notably, future deep space missions will likely require an automated psychotherapy option that can function independent of ground-based personnel, although automated psychotherapy options in the foreseeable future will likely involve a trained human therapist to some extent or another. Discussions of automated psychotherapy below are generally in reference to the more automated end of this treatment modality.

## Advantages of Automated Psychotherapy for Spaceflight

Automated psychotherapy has been widely discussed as a potentially popular method of delivering psychotherapy on Earth, as it allows treatments to be tailored to the client in a way that reduces the workload on experienced clinicians [36]. Cognitive behavioral therapy (CBT) is a common psychological treatment modality that is often modified into an automated psychotherapy course, usually referred to as internet CBT (iCBT). Here, it should be noted that the CBT umbrella is broad; many different components of other treatment modalities (eg, mindfulness-based treatments for anxiety disorders and exposure-based treatments for anxiety and trauma disorders) can fit under this umbrella, which is why CBT is often considered to be the gold standard of frontline psychological care [37]. A recent review has found that iCBT has been an effective treatment for a variety of psychopathologies, including depression, generalized anxiety disorder, panic disorder, obsessive compulsive disorder, posttraumatic stress disorder, adjustment disorder, bipolar disorder, and phobias, among others [38]. Recently, research has indicated that mindfulness-based interventions for stress, anxiety, depression, and other psychological disorders may be particularly useful in the spaceflight environment [39]. Given that automated psychotherapy has already been tested for mindfulness training, this may be a particularly useful function of this treatment modality [40]. In future spaceflight environments, automated psychotherapy may be the only form of treatment available to astronauts, especially during deep space missions [14]. Based on the empirical support for the use of automated psychotherapy for a wide range of psychopathologies, it is evident that automated psychotherapy has broad applicability and can be tailored to the individual in a flexible and supportive way [36]. In addition, the instantaneous nature of automated psychotherapy, and the lack of a need for a second party (the trained clinician), means that this treatment modality can easily be integrated into the astronaut’s schedule in a way that is convenient and accessible to them, reducing the barriers to treatment. Given that a lack of convenience is a commonly cited factor for not pursuing psychotherapy on Earth [36], reducing this barrier in a spaceflight environment is of the utmost importance.

In addition, automated psychotherapy provides a way for therapy to be conducted that does not require individuals to enter dual relationships (eg, both as professional colleagues and as therapist or client) [41]. In a spaceflight environment, the importance of this cannot be overstated. Given the known history of intracrew social tensions to quickly devolve the general morale and effectiveness of crews in space, any steps that can reduce the risk of social strain on space crews are steps worth taking. Similarly, the increased privacy of automated psychotherapy treatments can also protect the social reputation of crew members and reduce the likelihood of conflict. Given that some forms of automated psychotherapy allow for a client to undergo therapy without having to verbally speak, the opportunity for inadvertent or intentional eavesdropping from other crew members is significantly reduced. Accordingly, automated psychotherapy clients may feel more comfortable divulging their thoughts, feelings, and emotions in a therapeutic context without fear of their reports being overheard by other crew members. Notably, astronauts have previously cited a lack of privacy as a barrier to participating in psychosocial research while onboard the International Space Station [42], and these concerns can be expected to persist in a therapeutic environment.

Automated psychotherapy also creates the only pathway for therapy to occur between the client and another party not on the spacecraft that does not result in delayed communications. Given that an accumulation of stressors can threaten the therapeutic relationship and the overall effectiveness of psychotherapy [43], any steps that may reduce these stressors are worth pursuing. Automated psychotherapy offers a way to reduce the strain caused by delayed communications, protects the privacy of the client, reduces the risk of dual relationships, offers a tailored and customizable course of treatment, and has broad applicability for a wide range of psychopathologies. Accordingly, this treatment modality appears to be useful for future long-duration, deep space missions and may even offer advantages to crew members in low Earth orbit. As such, the usefulness of automated psychotherapy in a spaceflight environment should be further investigated.

## Drawbacks of Automated Psychotherapy for Spaceflight

However, automated psychotherapy is not impervious to all criticisms, and there are noteworthy concerns about this treatment modality. One potential detractor for automated psychotherapy is its apparently low treatment adherence rate compared with other psychotherapies; a meta-analysis of iCBT and face-to-face CBT adherence rates found that 84.7% of individuals receiving face-to-face CBT would complete their treatment, compared with 65.1% of individuals receiving iCBT [44]. Little data have been found regarding generalized predictors of treatment nonadherence for automated psychotherapy [45]. However, incorporating knowledge from the medical field more broadly may provide some insight to treatment nonadherence for automated psychotherapy. For example, the Medication Adherence Model provides a framework for understanding nonadherence to medication recommendations and treatments [46]. Within this model, it is thought that 2 different types of nonadherence may be seen: the intentional decision to miss a medication dose and the unintentional interruptions that can cause a medication dose to be missed. A core concept related to nonadherence is whether the patient shows purposeful actions to increase adherence, can demonstrate patterned behaviors to increase adherence, and are receptive to feedback to increase adherence. The Medication Adherence Model incorporates concepts from various cognitive and self-regulatory models of behavior to explain the processes involved in medication adherence [46]. Considering the framework provided by the Medication Adherence Model, it is possible that adherence to psychological therapies, and therefore, automated psychotherapies, may be impacted by similar factors. With regard to adherence to automated psychotherapy, it is possible that individuals who show purposeful actions to adhere to their treatment, can implement patterned behaviors to help adhere to their treatment, and are receptive to feedback regarding their treatment adherence may be able to complete automated psychotherapy modules at a higher rate than was previously found. The proposed ideal psychological profile for future deep space astronauts includes traits similar to these [12], which may indicate that future deep space astronauts will have a higher adherence rate to automated psychotherapies than the general population. However, an inclination to adhere to therapy provided by an automated psychotherapy system may be moderated by the astronaut’s belief that the information received from the system is accurate and helpful; if a client does not believe this to be the case, treatment adherence may be low. Accordingly, it is important to investigate how future deep space astronauts feel about the usefulness of these therapy solutions, and effort should be made to provide education surrounding the use of, and outcomes associated with, automated psychotherapy.

Automated psychotherapy may also be unsuitable for severe psychopathologies. Given that automated psychotherapy requires that an individual be able to self-monitor, adhere to their own treatment, and provide accurate assessments of their own thoughts and feelings, certain types of delirium, psychosis, or other severe psychopathologies may render an individual unable to use automated psychotherapy treatments. However, ruling out a treatment modality based on the severity of a diagnosis is common practice in therapeutic settings [47]. In the case that a crew member is experiencing a severe psychopathology, it is possible automated psychotherapies could be modified to incorporate another crew member to help provide the treatment. This, combined with the psychiatric formulary available to the crew, may offer a way to treat an individual who is far from Earth and is experiencing severe psychological symptoms.

It is also possible that while automated psychotherapy is intended to alleviate some of the uneasiness that an astronaut may feel about divulging sensitive mental health concerns to another person, this treatment modality may be unable to fully address this concern. For example, an astronaut may think that informing an automated psychotherapy program about their thoughts of depression, their anxiety surrounding a mission objective, or their feelings of isolation and loneliness will somehow be stored in an accessible personnel file or be relayed to mission planners on the ground. These concerns may prevent some astronauts from seeking help, and addressing these concerns will take deliberate education and planning to ensure that astronauts understand the full limits of confidentiality between themselves and the automated psychotherapist.

## First Examples of Automated Psychotherapy Options for Spaceflight

Numerous early, simplified examples of automated psychotherapy have been designed and tested for future long-duration, deep space missions. One such project aims to create a digital tool that allows astronauts to monitor their behavior, performance, and feelings; make small changes to their routines as recommended; and reduce stress in key areas to help reduce the risk of developing a psychopathology [48]. Similarly, an interactive media program is being developed to help astronauts cope with interpersonal conflict and depression while engaged in deep space missions [49]. This program was tested by a crew of 6 individuals who spent 8 months in group isolation in a space analog environment [50]. Overall, the space analog crew found the conflict and stress modules of the treatment to be particularly helpful, with one of the most cited areas of improvement being a desire to learn how to use these modules to help other crew members. In addition, there are various virtual and augmented reality methods for mitigating psychological demands under development and in various stages of testing. One such option is the Crew Interactive Mobile Companion, developed by IBM and Airbus Group and already tested on the International Space Station [51]. Crew Interactive Mobile Companion is designed as an artificial intelligence assistant for astronauts that is designed to offer them guidance on certain tasks and supply answers to technical questions. Overall, the development of automated psychotherapy for a spaceflight environment is still in its early stages. In addition, it should be noted that there is not public-facing information about what, if any, automated psychotherapy tools are currently available to astronauts in space. NASA and other space agencies should make a concerted effort to publish this information if it is available; if no automated psychotherapy tools are currently being used or tested in an actual spaceflight environment, efforts should be made to begin incorporating these as soon as possible.

## Variables Still to be Determined About Automated Psychotherapy for Spaceflight

Although much progress is being made with regard to developing automated psychotherapy treatments for long-duration, deep space missions, there are still key variables that have yet to be fully explored. More information is needed regarding adherence to automated psychotherapy, especially as it relates to the common personality profiles of astronaut crews. While the Medication Adherence Model offers general principles for treatment adherence [46], the applicability of this model to automated psychotherapy for astronauts has yet to be explored. There is also a need to investigate whether the usefulness of automated psychotherapy could expand the astronaut selection criteria. Currently, some of the astronaut selection criteria operate on a “rule-out” procedure, where certain medical, occupational, sociological, and psychological factors are searched for in order to disqualify a particular astronaut candidate [52]. Identifying the cognitive components most associated with treatment adherence to automated psychotherapy courses could help add to the existing “rule-in” procedures for astronaut selection (involving required skills or attributes for a given mission), wherein certain cognitive factors that would indicate that an astronaut candidate is more likely to respond to automated psychotherapy courses in the future are used when creating astronaut crew rosters. Given that it is almost expected that an astronaut will experience some level of psychological distress, discomfort, or disorder during future long-duration, deep space missions [53], perhaps it is worth searching for astronauts who best respond to psychological treatments, rather than restricting astronaut classes to only those who we believe are the most resilient against psychological disorders.

## Fitting Automated Psychotherapy Into Mental Health for Spaceflight More Broadly

Recent publications involving the mental health of the spaceflight environment more broadly have provided an excellent framework for understanding what our current knowledgebase is for space psychology and what research is still needed [41]. Notably, automated digital therapeutics has been recognized as a potentially useful psychological support tool for long-duration, deep space missions [54], indicating that multiple research teams have identified this as a helpful tool that should be developed. As research into digital therapeutics, a line of medical interventions that allow patients to interact with digital health technology in lieu of or in addition to medical professionals, accelerates, it is hoped that these technologies will continue to proliferate [55]. Automated psychotherapy can be conceptualized as an arm of digital therapeutics that has been proven to be particularly effective on Earth [34] and may drastically improve psychological outcomes for future astronauts that experience mental health difficulties.

## Conclusion

Automated psychotherapy offers a way for astronauts engaged in long-duration, deep space missions to receive empirically supported psychotherapy in a way that protects their privacy, reduces the risk of interpersonally straining dual relationships between crew members, and removes the difficulties presented by delayed communications. Although there are little data available regarding predictors of adherence to automated psychotherapy treatments, knowledge incorporated from the Medication Adherence Model suggests that the cognitive factors associated with reduced adherence to medical treatments may be less of a risk for astronauts due to the psychological selection criteria used by space agencies. The usefulness of automated psychotherapies for severe psychopathologies and unwilling participants has yet to be fully explored, but its overall efficacy for a broad range of psychopathologies that could be encountered during a long-duration, deep space mission indicates that automated psychotherapy could be a useful tool for safeguarding the mental health of future astronauts.

## Abbreviations

CBT: cognitive behavioral therapy
iCBT: internet cognitive behavioral therapy
