# Correction: Automated Psychotherapy in a Spaceflight Environment: Advantages, Drawbacks, and Unknowns

**DOI:** 10.2196/67671

**Published:** 2024-10-25

**Authors:** Logan Smith

**Affiliations:** 1 Oklahoma State University Stillwater, OK United States

In “Automated Psychotherapy in a Spaceflight Environment: Advantages, Drawbacks, and Unknowns” (Interact J Med Res 2024;13:e58803) the author noted one error.

The following sentence was erroneously included in the *Acknowledgements* section:

LS is now affiliated with Austin Peay State University and is no longer at Oklahoma State University.

This sentence has now been deleted, and the *Acknowledgments* section now reads as follows:

The author would like to thank Dr Nick Kanas for providing the inspiration for this review. This research did not receive any specific grant from funding agencies in the public, commercial, or not-for-profit sector.

The correction will appear in the online version of the paper on the JMIR Publications website on October 25, 2024, together with the publication of this correction notice. Because this was made after submission to PubMed, PubMed Central, and other full-text repositories, the corrected article has also been resubmitted to those repositories.

